# Supplementary material for: Dzip1 and Fam92 form a ciliary transition zone complex with cell type specific roles in Drosophila
Source: eLife. 2019 Dec 10;8:e49307. doi: 10.7554/eLife.49307 (PMC6904220; doi:10.7554/eLife.49307)
Supplement: Supplementary file 2. [file elife-49307-supp2.docx]

**Table S2. Primers used for plasmids, transgenic constructs and molecular characterisation**

| F-CBY1 | GGGGACAAGTTTGTACAAAAAAGCAGGCTTCATGCCTCTCTTTGGCAGCAT |
| --- | --- |
| R-CBY1 | GGGGACCACTTTGTACAAGAAAGCTGGGTCCTACTTCCTCCTGCGGTTGGTA |
| F-CbyGFP | TAATTC**GAATTC**CATGCCGCTCTTCAACAAG |
| R-CbyGFP | TAATTC**GGATCC**CGCTTTTCCTTTGGCTTCAGC, |
| F-HACby | TAATTC**GAATTC**CATGCCGCTCTTCAACAAG |
| F-HACby | TAATTC**GCGGCCGC**TCACTTTTCCTTTGGCTTC |
| F-Dzip1HA | TAATTC**GAATTC**TATGGGATTCAAGGGCAAATA |
| R-Dzip1HA | TAATTC**GCGGCCGC**TCATTTTAGGTTATCATCGCTG |
| F-Fam92HA | TAATTC**GAATTC**TATGTTTCGTCGTGGAAAACT |
| R-Fam92HA | TAATTC**TCTAGA**CTAGTGCTGGGGCACGTGGA |
| F-Dzip1GFPrep | TAATTC**GCGGCCGC**TTTCGCCCGAATCGCTG |
| R-Dzip1GFPrep | TAATTC**GTCGAC**TTTTAGGTTATCATCGCTGTCA- |
| F-Fam92GFPrep | TAATTC**AGATCT**ATACCAGCTCACGTCGTTG |
| R-Fam92GFPrep | TAATTC**GCGGCCGC**ATGTTCCTAATTCAATTTTATTTAAG |
| F-5’armDzip1 | taattcgcatgcCTGATCCAAATGCTTCAGATCC |
| R-5’armDzip1 | acgaagttatggtacctgcatatgTTCTCTGAATCTGAATCACCC |
| F-3’armDzip1 | acgaagttatcactagtaaagatctTCACATTCTGGACAGTGG |
| F-3’armDzip1 | taattcaggcctTACATTGAACAGGTGATAGG |
| F-Fam92KO | GCGCCATCTATGTACTTGCA |
| R-Fam92KO | CCTTAAACAGCTCCGAATCG |

**Bold letters represent cloning restriction sites**
